# Broadband luminescence in defect-engineered electrochemically produced porous Si/ZnO nanostructures

**DOI:** 10.1038/s41598-018-24684-6

**Published:** 2018-05-03

**Authors:** S. Dellis, N. Pliatsikas, N. Kalfagiannis, O. Lidor-Shalev, A. Papaderakis, G. Vourlias, S. Sotiropoulos, D. C. Koutsogeorgis, Y. Mastai, P. Patsalas

**Affiliations:** 1 Department of Physics, Aristotle University of Thessaloniki, Thessaloniki, GR-54124 Greece; 2 School of Science and Technology, Nottingham Trent University, Nottingham, NG11 8NS United Kingdom; 3 Department of Chemistry and the Institute of Nanotechnology, Bar-Ilan University, Ramat-Gan, Israel; 4 Department of Chemistry, Aristotle University of Thessaloniki, Thessaloniki, GR-54124 Greece

## Abstract

The fabrication, by an all electrochemical process, of porous Si/ZnO nanostructures with engineered structural defects, leading to strong and broadband deep level emission from ZnO, is presented. Such nanostructures are fabricated by a combination of metal-assisted chemical etching of Si and direct current electrodeposition of ZnO. It makes the whole fabrication process low-cost, compatible with Complementary Metal-Oxide Semiconductor technology, scalable and easily industrialised. The photoluminescence spectra of the porous Si/ZnO nanostructures reveal a correlation between the lineshape, as well as the strength of the emission, with the morphology of the underlying porous Si, that control the induced defects in the ZnO. Appropriate fabrication conditions of the porous Si lead to exceptionally bright Gaussian-type emission that covers almost the entire visible spectrum, indicating that porous Si/ZnO nanostructures could be a cornerstone material towards white-light-emitting devices.

## Introduction

White light emission is an important technical field with extremely high environmental, economic, and social impact. The current state-of-the-art in white solid state lighting is based on two device architectures^1^. The first one is based on an assembly of three light emitting diodes (LED), which emit red, green, and blue light, in combination with a diffuser screen. The second architecture combines a short wavelength LED (either UV or blue) with a yellow emitting phosphor material in a single assembly. Therefore, there is an increasing demand for materials that can emit directly broadband white light^2,3^. One of the most well-established luminescent materials is ZnO, which exhibits both electroluminescence^4,5^ and photoluminescence^6,7^. Its direct bandgap leads to strong UV emission, which is known as near-band edge (NBE) emission^6–11^. ZnO may also exhibit broadband emission in the visible range, which is usually centered around the green^12–14^ but may be extended to yellow and orange^14–16^, as well. This visible emission originates from deep-level states into the gap of ZnO, which are associated with defects such as O vacancies among others^7,17–20^, and thus it is called deep-level emission (DLE). Therefore, and given the variety of luminescent centers in defective ZnO, a defect-engineered ZnO may be a very promising candidate towards white light applications. The importance of ZnO, being an oxide, is further enhanced by its growth in ambient air without the need of vacuum^21^. Another well-known luminescent material is porous Si (pSi); pure pSi’s luminescence is theoretically predicted to be in the range of blue or shorter wavelengths due to quantum confinement^22^. However, experiments have shown that the fabrication of pSi emitting blue or white light is rather difficult, due to the formation of surface Si-O bonds that result in stronger red/yellow luminescence^23,24^. Even for the case of pSi nanocrystals smaller than 2.0 nm, where the quantum confinement would be expected to maximize the blue or white emission, the red/yellow emission, due to the Si-O bonds, prevails for geometrical reasons, as the surface to volume ratio of such nanocrystals is also maximized. Consequently, pSi in most cases exhibits strong yellow/red emission instead of blue^25^, making it unsuitable for white light applications on its own merit. The coupling of the pSi surface with a luminescent material that presents a blue/green emission has been proposed as a route towards white light emission^26–30^. Thus, combined pSi/ZnO nanostructures (NS) were studied by many groups as potential candidates for white light emission via optical pumping; pSi/ZnO can be also a promising candidate for electrically-pumped devices given that *p*-type Si is combined with the intrinsically n-type ZnO^31,32^. In particular, *R.G. Singh et al*. reported the photoluminescence properties of pSi/ZnO NS. An intense broadband emission was manifested by combining the blue-green emission from ZnO and orange-red from pSi^26^. Two years later, the same research group attempted to explain this broadband emission based on electron tunneling between the interface of ZnO and pSi through a siloxane structure^27^. In both papers, the ZnO structures were prepared by sol-gel deposition. A different fabrication approach was suggested by *E. Kayahan* with the combination of RF-magnetron sputtering and thermal annealing of the NS^28^. The observed broadband light emission was attempted to be explained by using an oxygen-bonding model in the pSi and native defects in ZnO. The origin of the broadband light emission was investigated by *O. Marin et al*. that identified the luminescence centres in the pSi/ZnO NS^29^. Finally, the PL emission of the NS has been correlated with the porosity of the pSi substrate^30^.

Sputtering^28^, Sol-Gel^26^ and Vapor Transport Deposition (VTD) techniques^29^ have been used, so far, for the preparation of pSi/ZnO NS. However, all of the aforementioned growth routes exhibit substantial drawbacks; in particular, sol-gel is incompatible with Complementary Metal Oxide Semiconductor (CMOS) technology, and consequently its products cannot be integrated in mainstream micro- and optoelectronic devices, while sputter deposition and VTD suffer from limited potential to grow conformal films into the pores, and as a result, the nanostructuring in sputtered or VTD ZnO stems exclusively from the underlying pSi^28^. Finally, most of the studied techniques, so far, are based on vacuum technology, which imposes significant limitations in terms of cost and scalability.

**Figure 1:**
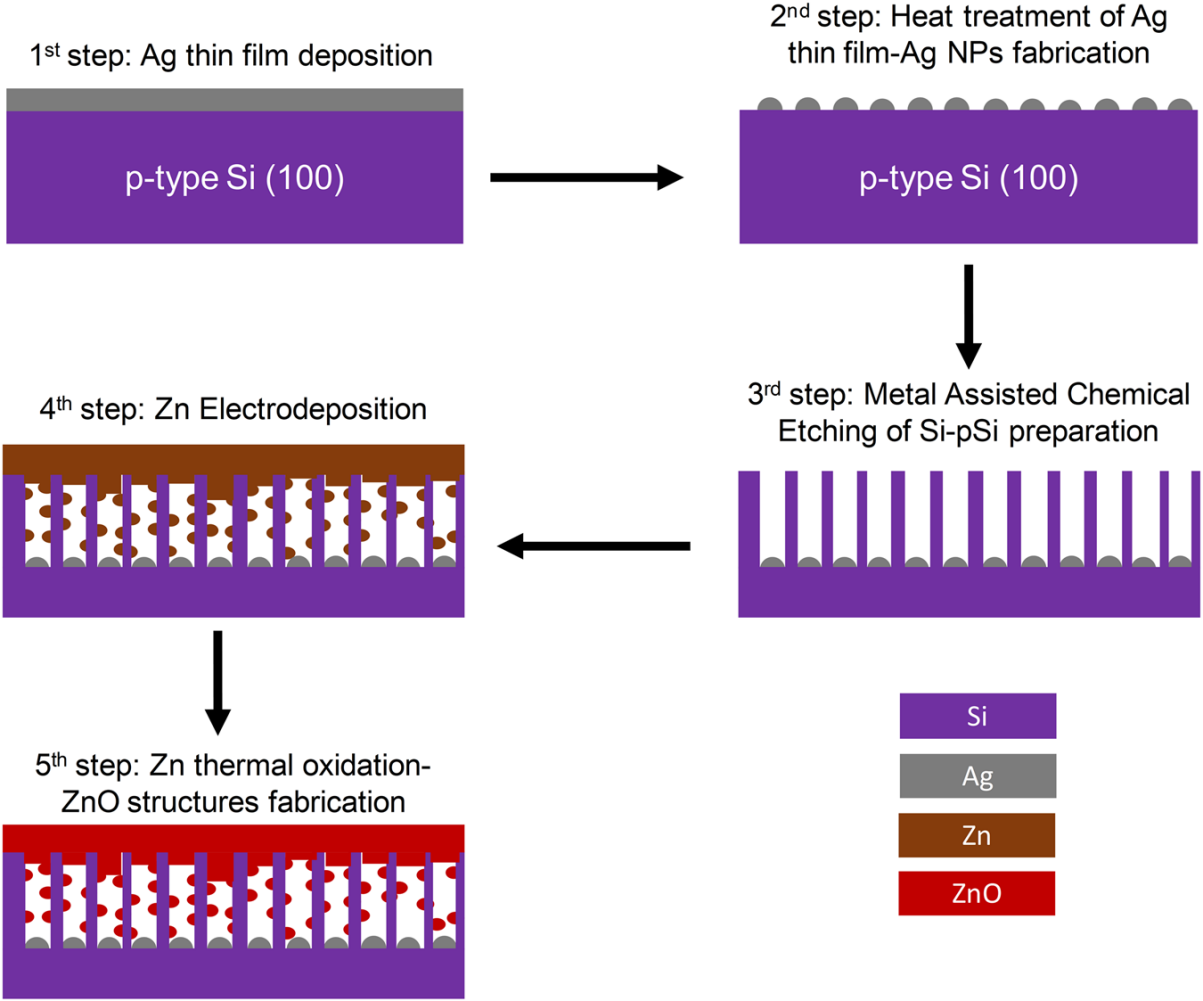
Schematic of the presented process for the production of pSi/ZnO NS emitting broadband light in the visible spectrum.

In this work, we propose an entirely vacuum-free fabrication process for pSi/ZnO NS with exceptional potential for defect-engineered ZnO towards white lighting applications. The proposed method comprises an all-electrochemical process, a facile and inexpensive way of fabrication, which can give well-controlled and reproducible results and is superior to the current state-of-the-art deposition techniques in terms of simplicity, scale-up potential and versatility on the design and performance of pSi/ZnO NS (the fabrication steps of the samples are presented in Fig. 1). We also present the PL properties of such NS and the effect of the pSi substrate morphology on the emission properties; in particular, we identify the different luminescent centers and we correlate the emission at various visible colors with the crystallographic characteristics of the electrodeposited Zn. This method qualifies as an inexpensive, fast, scalable and simple way towards the fabrication of luminescent NS, with PL that can be easily enhanced and manipulated, hence it can be a very promising innovation towards the fabrication of white light emitting devices.

## Results and Discussion

pSi substrates were fabricated using an electroless etching process that requires three individual steps: Firstly, a 5–15 nm Ag thin film was deposited on top of a *p*-type Si (100) wafer (1–10 Ohm·cm) by magnetron sputtering; note that the use of sputtering for the deposition of silver is not essential, as similar silver ultrathin films can be deposited by a variety of vacuum-free techniques, such as electroless deposition^33,34^, gravure printing^35^, and inkjet printing^36^. Secondly, the Si/Ag structure was heated to 300 °C for 30 s, on a hot plate. This step formed Ag nanoparticles (NPs) on top of the Si substrate due to dewetting of Ag^33^. Their size strongly depends on the initial thickness of the Ag thin film. The third step, during which the pores are formed, is the immersion of the Si/Ag NPs structure in an aqueous solution of 4.6% HF and 0.6% H_2_O_2_ (v/v) for 30 min at room temperature (RT)^37^. The diameter of the pores, as well as the porosity (the percentage of the surface area covered by the pores) of the pSi substrates, were both directly correlated to the size and the population of the Ag NPs, respectively (see the Supplemental Information, Fig. S1). Furthermore, the length of the pores can be controlled by the duration of the etching process. The prepared pSi substrates were used as the working electrode for the electrodeposition of metallic Zn; a 20 V bias was applied between the pSi and the counter electrode (graphite rods) in an aqueous solution of 5 mM ZnSO_4_∙7H_2_O and 0.1 M NaCl at room temperature (RT)^38^. The use of high voltage (20 V) stems from the *p*-type character of Si as its high resistivity makes it a blocking contact in the electrodeposition process^39^. The electrodeposited pSi/Zn samples were then thermally oxidized in a quartz tube at 500 °C for 60 min in atmospheric air to form pSi/ZnO NS. This methodology could also show great potential for the fabrication of intrinsically *n*-type ZnO NS based LEDs on top of *p*-type substrates, as an alternative method of fabricating ZnO *n-p* heterojunctions, which presents both difficulty in the preparation and low efficiency^4,40^.

**Figure 2:**
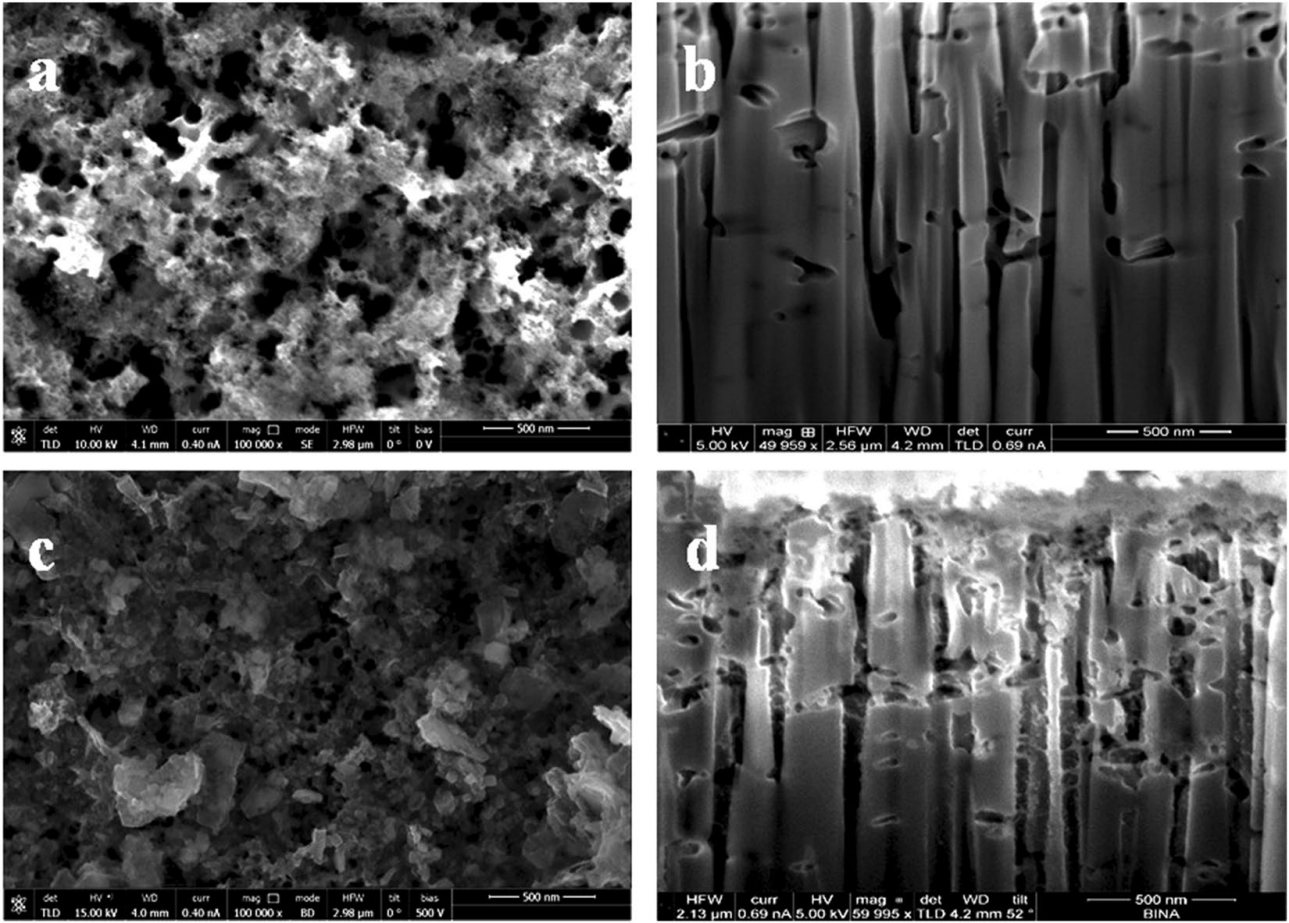
(**a**) Plane-view SEM image of the pSi substrate, (**b**) cross-section SEM image of the pSi substrate after focused ion beam etching, (**c**) plane-view image of the pSi/ZnO NS and (**d**) cross-section image of the pSi/ZnO NS.

The pores’ formation process in Si is a cycle of oxidation and dissolution of silicon oxide that is in contact with the metal NPs^41,42^. The NPs initially are hemmed in the amorphous native SiO_2_ so they move in random directions and give a sponge-like morphology to the pSi surface, as shown in Fig. 2a. However, after the native oxide is completely etched a different mechanism unfolds: the nanoparticles find their way towards the Si monocrystalline substrate and start etching it preferentially across the (100) orientation, which in our case is perpendicular to the surface of the substrate (Fig. 2b). The net result of this process is the fabrication of very well aligned and directional pores into the bulk volume of the Si substrate. The average pore diameter is dictated by the Ag nanoparticle size (for more details refer to the Supplemental Information, Figs S1 and S2 and the relevant text), which also defines the Localized Surface Plasmon Resonance (LSPR) spectral position of the nanoparticles before the metal-assisted etching step^33^.

The DC electrodeposition of Zn and the subsequent thermal oxidation, resulted in the coverage of the pSi surface by the nanostructured spongy ZnO, as shown in Fig. 2c (for high-resolution images refer to the Supplemental Information, Fig. S3). The fabricated ZnO NS seem not to cover the Si pores in their entirety, as shown in Fig. 2d (and Fig. S3b in higher resolution); in addition, the volume fraction of the ZnO deposited on the surface of the pSi is much higher compared to the pores. This is an indication of preferential deposition of Zn on the outer surface of the substrate compared to deposition on the pore walls, due to ohmic losses and mass transport limitations, that results in blockage of the pores^43^. Consequently, the major effect of the pSi substrate is not the formation of ZnO into the pores itself, but the hindering of extended growth of ZnO along the x-y surface plane that results in the formation of ZnO grains, whose majority sizes are 100 nm or less (shown in Fig. S3a) as well as the incorporation of different point defects into the produced ZnO compared to the deposition on polished Si wafers, as we will discuss in more detail later in this work.

**Figure 3:**
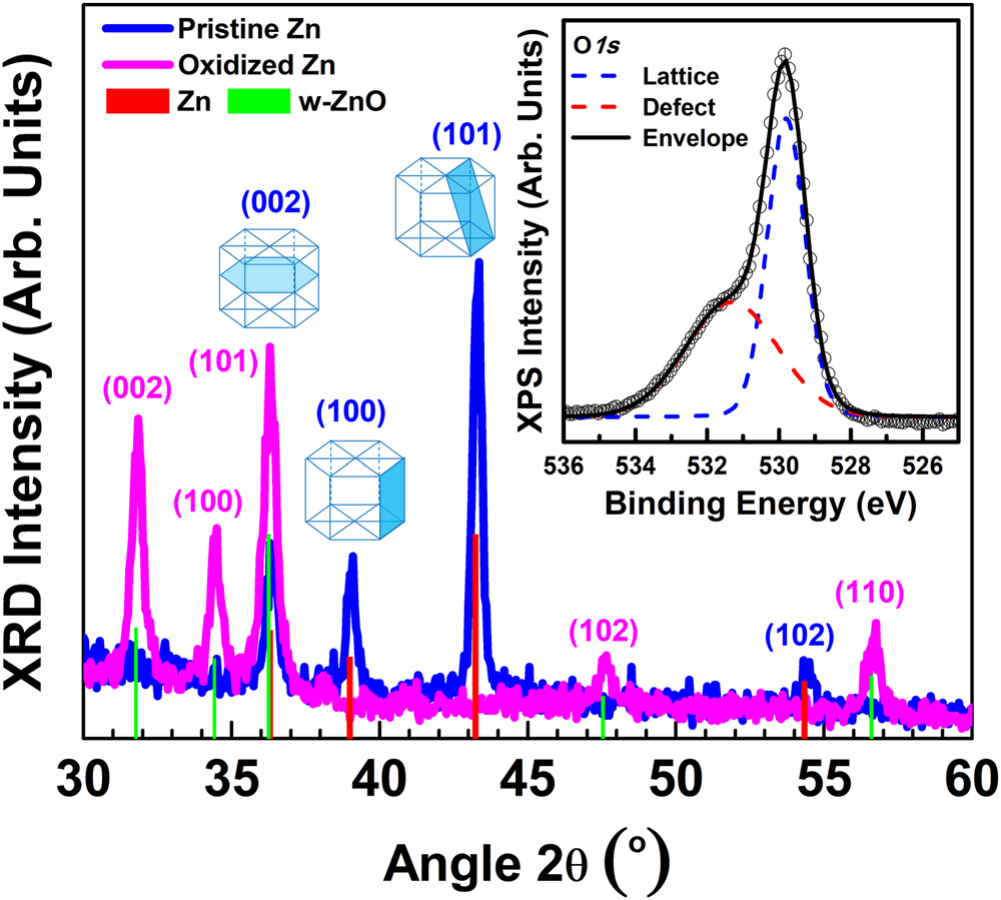
XRD patterns of the sample with the smallest pores before thermal oxidation (pSi/Zn, blue line and Miller indices) and after thermal oxidation (pSi/ZnO, magenta line and Miller indices); the dominant crystal planes of metallic hcp Zn are also depicted for clarity. The inset presents the O1s core level XPS spectrum after thermal oxidation that reveals the existence of a substantial number of structural defects.

Typical XRD patterns from the sample with the smallest pores in pSi before and after thermal oxidation are presented in Fig. 3. The XRD pattern of the NS immediately after the electrodeposition reveals that only metallic *hcp* Zn exists with preferential (101) orientation; this preferential orientation of Zn is associated with the porosity of pSi and is essential for the luminescence of the pSi/ZnO NS, as we will discuss in more detail later. After thermal oxidation, all the peaks of metallic Zn disappeared and only peaks corresponding to wurtzite-type ZnO (w-ZnO) were present. This was confirmed by the chemical composition of the samples (both on their surface, as well as at 10 and 20 nm below the surface), which was investigated by XPS. The survey XPS spectra from the pSi/ZnO NS with the smallest pores at various depths are shown in the (Supplemental Information Fig. S4). In the XPS spectrum of the pSi/ZnO NS’s surface, the peaks of C *1s*, Zn *2p*_*3/2*_, Zn *2p*_*1/2*_ and O *1s* are clearly observed, from which an [O]/[Zn] ratio exceeding 1 is determined, however, it is still in the range of stoichiometry that w-ZnO is observed. The XPS analysis also demonstrates the high chemical purity of the produced ZnO samples as no impurity elements (such as Si and Ag from the substrate, or C from the counter electrode, or S, Na and Cl from the electrodeposition solution) were detected into the films whatsoever. As the survey scan spectra, cannot identify the chemical state of the detected O (i.e. if it is exclusively lattice or defect O into ZnO or if it is adsorbed on the high effective surface of the spongy ZnO), we consider the O *1 s* core level spectra as presented in the inset of Fig. 3. Indeed, the O *1 s* peak can be deconvoluted in two peaks, one located at 529.8 eV, corresponding to O^2−^ in wurtzite lattice sites and another one at 531.3 eV, correlated with the O^2−^ in the oxygen deficient regions of ZnO^44^. The area percentages of these curves were calculated to be 52% and 48% for lattice and defect sites, respectively.

**Figure 4:**
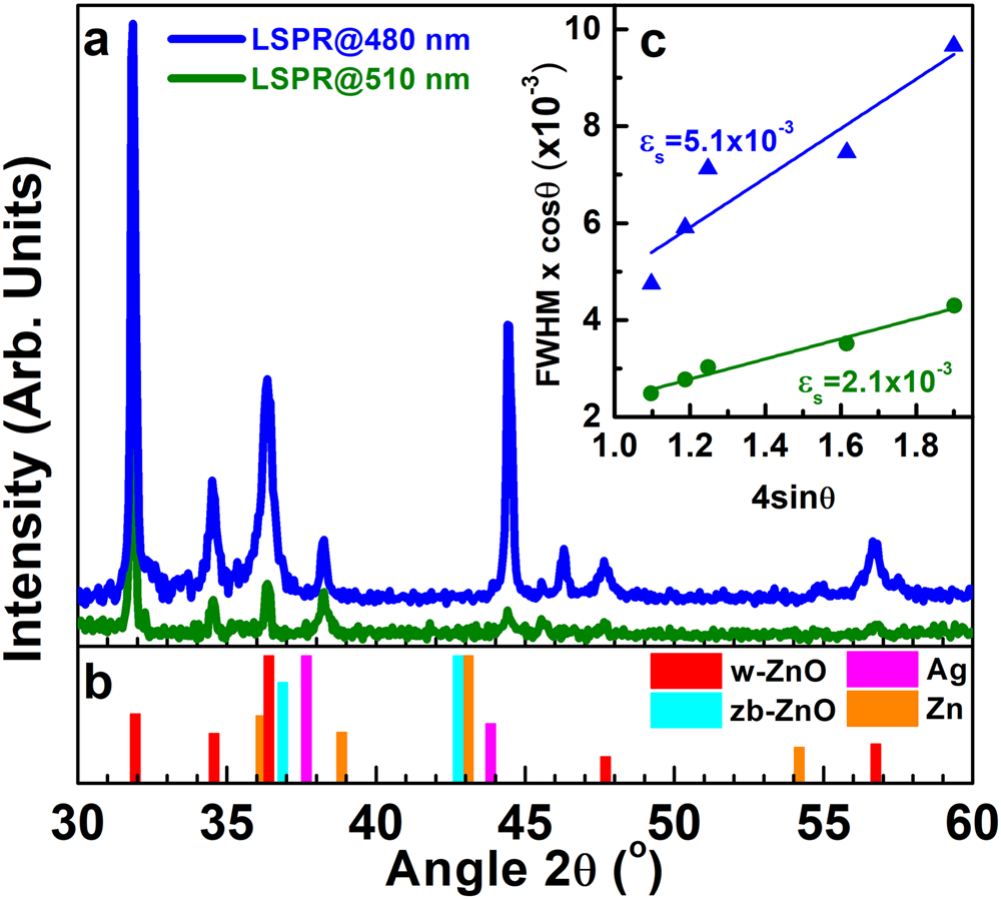
(**a**) XRD patterns of two pSi/ZnO samples fabricated using Ag NP templates of two different sizes and LSPR wavelengths (480 and 510 nm), (**b**) the powder diffraction reference patterns for w-ZnO, zb-ZnO, Ag and metallic Zn, (**c**) Williamson-Hall peak profile analysis for the determination of the micro-strain εs, which is associated with the structural defect density.

Quantitative information regarding the structural quality of ZnO films and correlations of the micro-strain ε_s_ and their correlation to the pore size of pSi were derived from XRD analysis. The micro-strain ε_s_ is associated with the structural defects of ZnO and the mismatch of the pSi/ZnO nanostructure interface, while the pore size is dictated by the size (and consequently the LSPR spectral position^33,45^) of the Ag NPs used for the Si etching. Indeed, Fig. 4 shows the XRD patterns of two different pSi/ZnO samples fabricated with the use of two different pSi substrates (Fig. 4a), as well as the reference XRD patterns of polycrystalline Zn, hexagonal wurtzite-type w-ZnO, cubic zincblende-type zb-ZnO, and Ag and metallic Zn powders (Fig. 4b). The broadening (full width at half maximum-FWHM) of each XRD peak, associated with w-ZnO, *vs* 4sinθ (where θ is the Bragg angle of each peak) are presented in the inset graph (Fig. 4c). The XRD patterns were quantified by employing the Williamson-Hall analysis^46^ to the profiles of the w-ZnO peaks according to which:

1 $$FWHM\cdot \,\cos \,\theta =\lambda /D+4{\varepsilon }_{s}\cdot \,\sin \,\theta $$

In Eq. 1, *FWHM* and *θ* are the broadening and angular position of each XRD peak of w-ZnO, *λ* is the X-ray wavelength (0.154 nm in our case) and *D* is the average crystallite size. The results of this analysis show that the smaller pore size in the pSi substrate (pSi prepared with the use of smaller Ag NPs) leads to higher ε_s_, *i.e*. higher number of structural defects, which give rise to the DLE.

**Figure 5:**
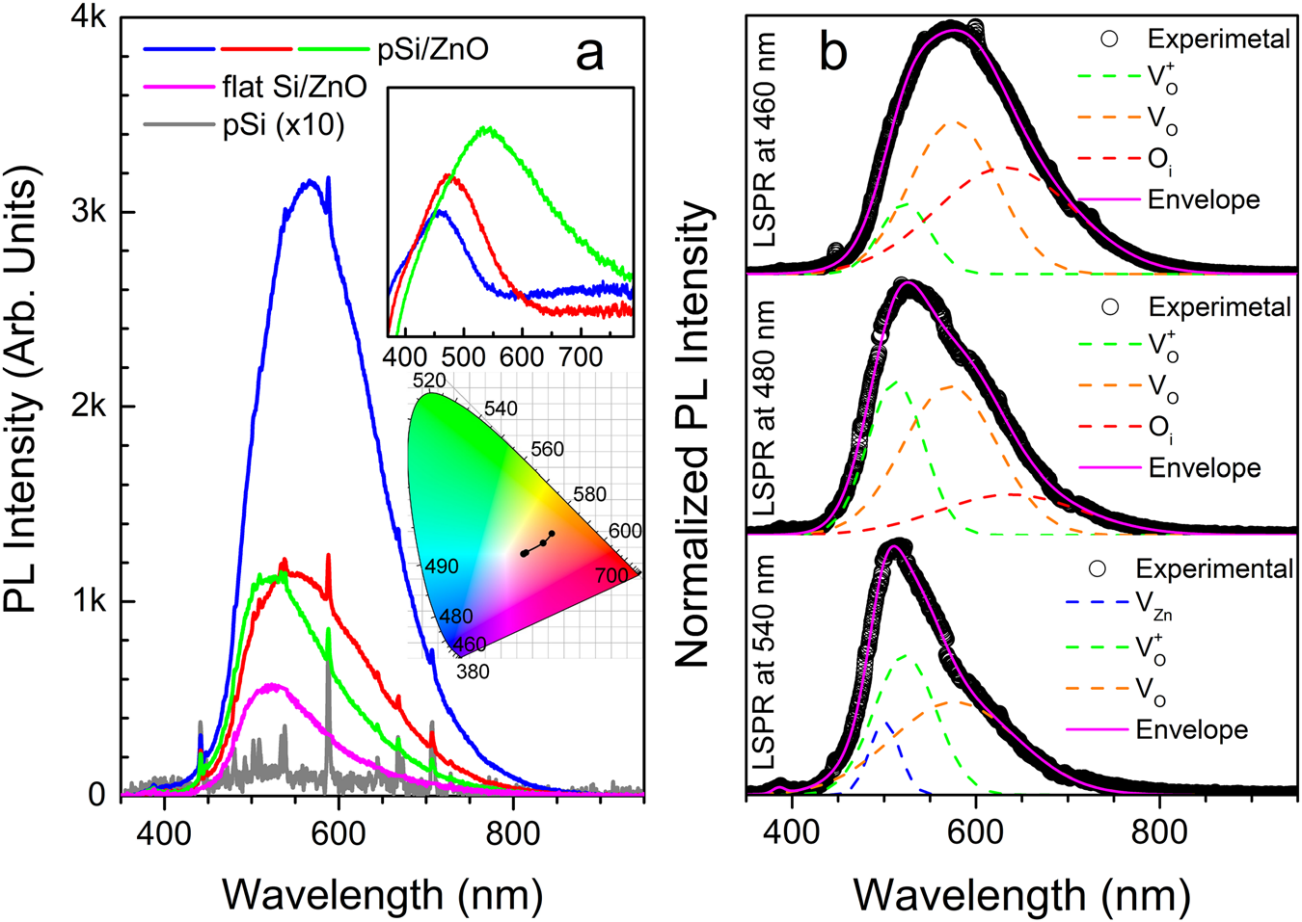
(**a**) PL spectra of the pSi/ZnO samples fabricated using pSi substrates with different pore sizes. The upper inset presents the reflectivity spectra of the Ag NPs used for the Si etching, in matching colors; thus, the finest and widest pores correspond to the blue and green PL and reflectivity spectra, respectively, while the red spectra are the intermediate case. Also shown are the PL spectra of bare pSi after thermal oxidation at 500 °C for 1 h ( × 10) and of a ZnO thin film electrodeposited on top of a flat Si with identical conditions to the pSi/ZnO; the lower inset shows the color coordinates of the emitted light from pSi/ZnO within the CIE scheme. (**b**) Deconvolution of the normalized PL spectra of pSi/ZnO to contributions originating from various point defects in Zn.

The effects of the aforementioned structural features to the PL spectra of pSi/ZnO NSs are presented in Fig. 5, which shows that the higher number of structural defects leads to broader DLE emission. In particular, Fig. 5 shows the room-temperature PL emission of three samples, fabricated with the use of three different pSi substrates (red, green, blue lines). The deposition conditions of the ZnO were the same for all these samples, and only the morphology of the underlying pSi varied. The inset of Fig. 5a depicts the reflectivity spectra of the Ag NPs that were used for the preparation of the corresponding pSi substrates (as shown in Fig. S1 the Ag particles size used for the etching has the same lateral size with the produced pores). In Fig. 5 the PL spectrum of a bare pSi after thermal oxidation at 500 °C is also shown, and it demonstrates that the pSi itself does not provide any broadband PL and, therefore, the observed broadband PL is assigned exclusively to ZnO. The PL spectra of the pSi/ZnO exhibit a blueshift and become sharper with increasing the Ag nanoparticle size, and consequently Si pore size, as well, gradually approaching the spectral profile and strength of the DLE emission of ZnO deposited on polished Si wafers (Fig. 5, magenta line). A more detailed view of the line shape and the spectral shifts of the PL spectra can be seen in the normalized form presented in Fig. S5 in the Supplemental Information. The PL spectrum of the pSi/ZnO NS with the finest pores (Fig. 5, blue line) has nearly perfect Gaussian shape that extends in the entire visible spectrum; in addition, the PL emission of pSi/ZnO with the finest pores has threefold and sixfold highest intensity compared to pSi/ZnO with large pores and ZnO on polished Si, respectively, due to the increase of the structural defects with reducing pore size, as shown by the XRD analysis. The detected visible emission of all samples is dominated by the ZnO and not pSi, apart from the very sharp lines at 537, 588, 667, 708 nm, which are also observed in bare pSi after thermal oxidation (Fig. 5a, grey line). Figure 5a also shows that the broadband emission of pSi itself is very weak and its intensity is orders of magnitude smaller than the strongest DLE observed in the pSi/ZnO NS (see Fig. S6, as well).

The DLE emission of ZnO has been assigned to several structural defects that induce luminescent centres within its bandgap. Oxygen vacancies are a well-studied type of such defects that lead to green emission^14,47^. Other structural defects can also create different energy levels inside the ZnO bandgap emitting light in several wavelengths, thus extending the PL emission range^13,48,49^. In an effort to quantify the PL spectra and identify the source of the broadband emission for the smallest pore sizes of pSi/ZnO, we deconvoluted the PL spectra into individual Gaussian contributions that are associated with specific defects; although theoretical works have shown that V_O_ is a deep negative-U donor that has only one state in the band gap^50^ there are plenty of experimental works reporting a variety of defects in ZnO that result in various defect states in the band gap and light emission from them^7,17,18,20,49,51–57^. Following the works of *Barbagiovanni et al*.^57,58^, we identify four Gaussian curves located at approximately 500 nm (~2.5 eV), 530 nm (~2.3 eV), 580 nm (2.1 eV) and 630 nm (~1.9 eV) attributed to Zinc vacancies (V_Zn_), ionized oxygen vacancies (V_O_^+^), neutral oxygen vacancies (V_O_), and oxygen interstitials (O_i_), respectively^57,59^. The concentration of each defect varies leading to slightly different lineshapes, as shown in Fig. 5b. All spectra are dominated by the contributions of the V_O_^+^ and V_O_, which are the source of the well-known green-yellow luminescence. As the pores of the underlying pSi become finer, the red luminescence due to O_i_ is emerging; for the finest pores (<50 nm, Fig. S1) this red contribution balances the green-yellow luminescence resulting into the bright broadband light emission (Fig. 5 and lower inset). The origin of the O_i_ in pSi/ZnO with fine pores can be well explained by considering the morphology of the electrodeposited Zn film before the final oxidation step as revealed by XRD (see Supplemental Information, Fig. S7). Indeed, the electro-deposited Zn on polished Si wafer (Fig. S7, blue line), where there is no contribution whatsoever of O_i_ to the PL spectra (Fig. 5a), is predominantly grown along the [001] direction [*i.e*. the (002) close-packing planes are parallel to the surface] and exhibits sharper XRD features (due to larger grain size and less structural defects) than the Zn grown on pSi (Fig. S7, red line), which is predominantly grown with the side planes (100) and (101) parallel to the surface. Consequently, during oxidation the close packing of the (002) planes of the large Zn crystals electrodeposited on polished Si hinders the diffusion of excess oxygen into interstitial sites. On the contrary, oxygen may easily penetrate into interstitial sites via the open side planes [(100) and (101)], which are parallel to the surface of the electrodeposited Zn on pSi giving rise to the red luminescence that complements the universally existing green-yellow luminescence of ZnO and thus broadband light emission is created.

## Conclusions

In conclusion, pSi substrates of varying pore sizes were fabricated by an electroless etching technique that can provide high control on the volume fraction, length and morphology of the pores. These substrates were used for the deposition of ZnO nanostructures with engineered defects using a low-cost, CMOS compatible and scalable process based on DC electrodeposition and subsequent thermal oxidation of Zn. The PL emission of these nanostructures is strongly dependent on the underlying pSi substrate and can be designed to produce a near Gaussian-shape emission that covers the entire visible spectrum whilst presenting exceptional brightness. The use of pSi substrates is essential for hindering the growth of ZnO along high-packing (002) planes and thus promoting the incorporation of oxygen interstitials during oxidation of Zn, giving rise to the otherwise missing red component of ZnO’s PL emission. These results clearly demonstrate that pSi/ZnO nanostructures could be a very competitive platform towards the preparation of low-cost white-light emitting devices.

## Methods

The crystal structure of the samples was investigated by XRD in Bragg-Brentano geometry using a Rigaku Ultima^+^ instrument equipped with a Cu anode and a graphite monochromator. XPS spectra were acquired in a KRATOS Axis Ultra DLD system equipped with a monochromated Al K_α_ X-ray source. High-resolution Scanning electron microscopy (HR-SEM) images were obtained with Magellan XHR 400 L FE-SEM – FEI instrument (FEI, USA) at acceleration voltages of 15 and 20 kV. Cross section images of the samples were taken using a Focused Ion Beam (FIB) Helios 600 scanning electron microscope system (FEI).

PL excitation was performed using a CW HeCd laser (325 nm) with a circular 5 mm beam spot incident on the film surface. Typical incident beam power for the PL characterization was 9.2 mW cm^−2^. The collection of the emitted light took place through an appropriate optical fiber to an Ocean Optics S2000 spectrometer, which is responsive from 200–1100 nm. All spectra were acquired with the same integration time, laser focus conditions and light collection geometry to enable quantitative comparisons.

### Data Availability Statement

All data generated and analysed during this study are included in this published article (and its Supplementary Information file).

## Electronic supplementary material

Supplementary Information
