# Supplementary material for: Broadband luminescence in defect-engineered electrochemically produced porous Si/ZnO nanostructures
Source: Sci Rep. 2018 May 3;8:6988. doi: 10.1038/s41598-018-24684-6 (PMC5934408; doi:10.1038/s41598-018-24684-6)
Supplement: Supplementary file 1 — Supplementary Information [file 41598_2018_24684_MOESM1_ESM.docx]

**Broadband luminescence in defect-engineered electrochemically produced porous Si/ZnO nanostructures**

S. Dellis^1^, N. Pliatsikas^1^, N. Kalfagiannis^2^, O. Lidor-Shalev^3^, A. Papaderakis^4^, G. Vourlias^1^, S. Sotiropoulos^4^, D.C. Koutsogeorgis^2^, Y. Mastai^3^, and P. Patsalas^1, *^

^1^Department of Physics, Aristotle University of Thessaloniki, Thessaloniki, GR-54124, Greece

^2^School of Science and Technology, Nottingham Trent University, Nottingham, NG11 8NS, United Kingdom

^3^Department of Chemistry and the Institute of Nanotechnology, Bar-Ilan University, Ramat-Gan, Israel

^4^Department of Chemistry, Aristotle University of Thessaloniki, Thessaloniki, GR-54124, Greece

[*ppats@physics.auth.gr](mailto:*ppats@physics.auth.gr)

**On-line Supplemental Information**


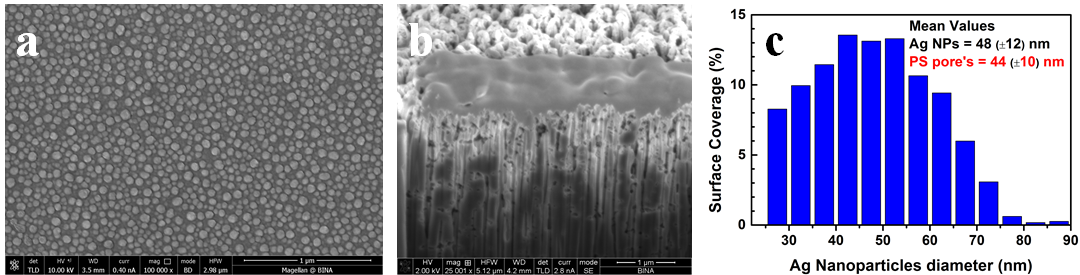


**Fig. S1.** (a) Plane-view SEM image of Si covered by Ag NPs which were prepared by heating a 5nm Ag thin film on a hot plate at to 300 ^o^C for 30 sec, (b) cross-section view of pSi fabricated with metal-assisted chemical etching, (c) the size distribution of the Ag NPs; the mean value and standard deviation of the Ag NPs’ and pSi pores’ sizes were calculated by quantitative analysis of the SEM images.

In the metal-assisted chemical etching process, the diameter of the pSi pores is dictated by the diameter of the Ag NPs that were used for the pSi fabrication. This is vividly illustrated in Fig. S1, in which the Ag NPs (Fig. S1a) and the respective pSi (Fig. S1b) are presented. The size distribution of the Ag NPs is presented in the Fig. S1c. The mean value of the particle diameter has been calculated equal to 48 nm with a standard deviation of 12 nm. The mean pores’ diameter of the pSi, calculated from the Fig. S1b cross-section photo is 44 nm with a standard deviation of 10 nm. The two values are in good agreement proving the direct correlation between pores’ and nanoparticles’ sizes. The different Ag nanoparticle sizes manifest by the different position of their LSPR according to Ref. 31; smaller particle sizes result in LSPR at shorter wavelengths. Indeed, Fig. S2 demonstrates the refinement of pSi pores for the cases of etching by Ag nanoparticles of reducing sizes and LSPR wavelengths.

**Fig. S2:** Cross-section SEM images of bare pSi produced by etching using Ag NPs of different sizes and LSPR wavelengths: (a) LSPR@690 nm, (b) LSPR@580 nm, (c) LSPR@510 nm.

ZnO structures are formed on top of pSi substrates. These structures are agglomerates of small grains with sizes smaller than 100 nm. Most of the surface after the electrodeposition of Zn and its thermal oxidation is covered by such ZnO nanostructures. On the other hand, some pores of the pSi substrates seem to be unfilled (*e.g.* see region A in Fig. S3) while most pores are filled with ZnO nanostructures (see regions B, C, and D in Fig. S3).

**Fig. S3:** High-magnification images of Figs. 1c, d that vividly demonstrate the lateral grain size of ZnO and the pSi pores filling by ZnO nanoparticles.

**Fig. S4:** Survey XPS spectra of pSi/ZnO with the smallest pores from the un-etched surface and after etching of 10 nm and 20 nm. In the inset, the atomic concentrations of Zn, O and C *vs.* depth are presented.

The chemical composition of the surface of pSi/ZnO samples after the thermal oxidation, as well as after 10 nm and 20 nm etching, were measured by XPS, which detected exclusively Zn, O and C (Fig. S4). The C *1s* peak is completely suppressed after the etching procedure, proving that the existence of C on the surface has exclusively the form of adventitious carbon due to exposure to the ambient. No other peaks, which may correspond to any element from the salts used in the electrodeposition, or from Ag that was used in the Si etching process are present, proving the high chemical purity of ZnO achieved.

**
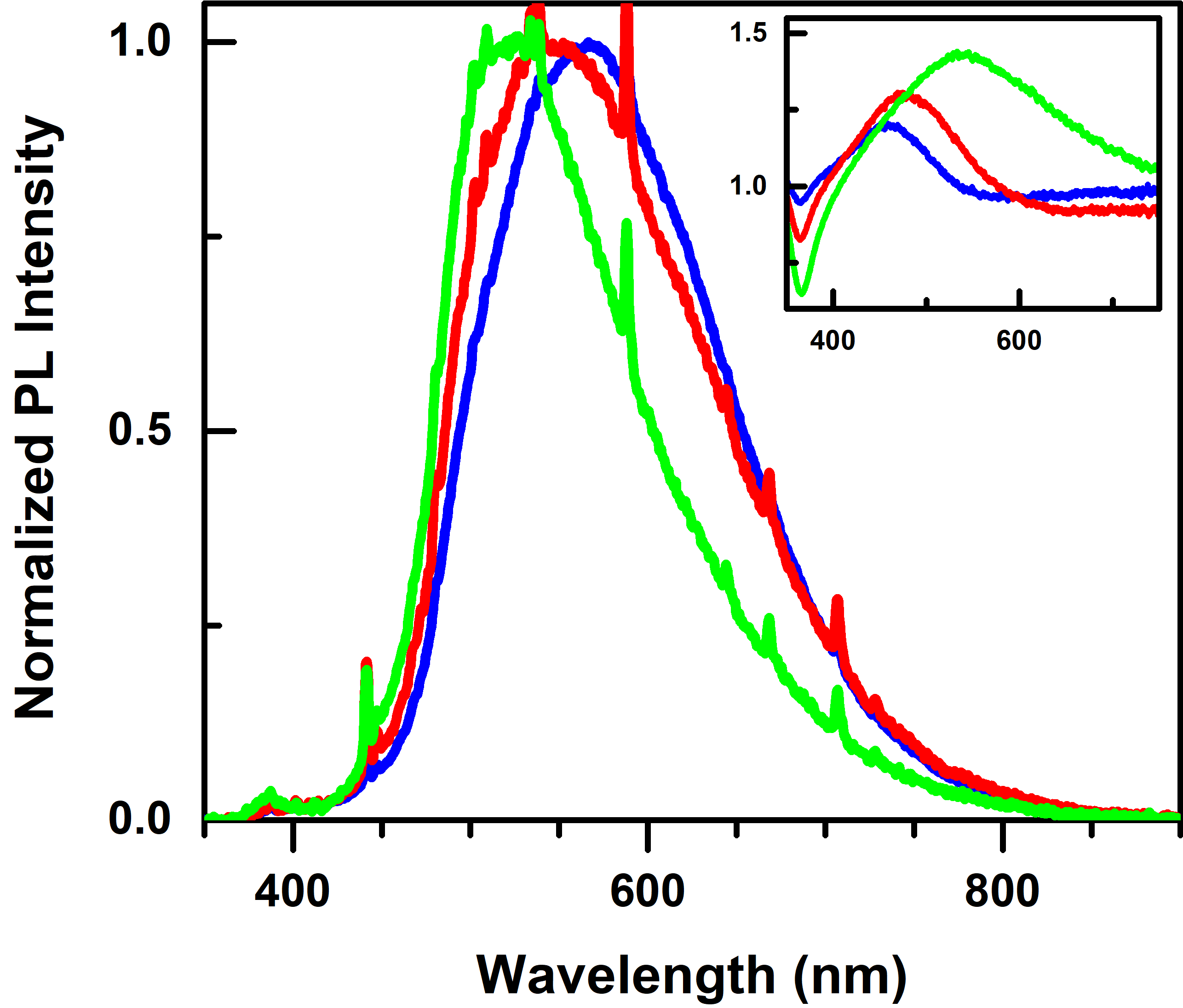
**

**Figure S5.** Normalized PL spectra of pSi/ZnO NS that were fabricated with the use of pSi of varying pore sizes, where the spectral shifts are vividly illustrated. The inset presents the reflectivity spectra of the Ag NPs used for the Si etching, in matching colors.


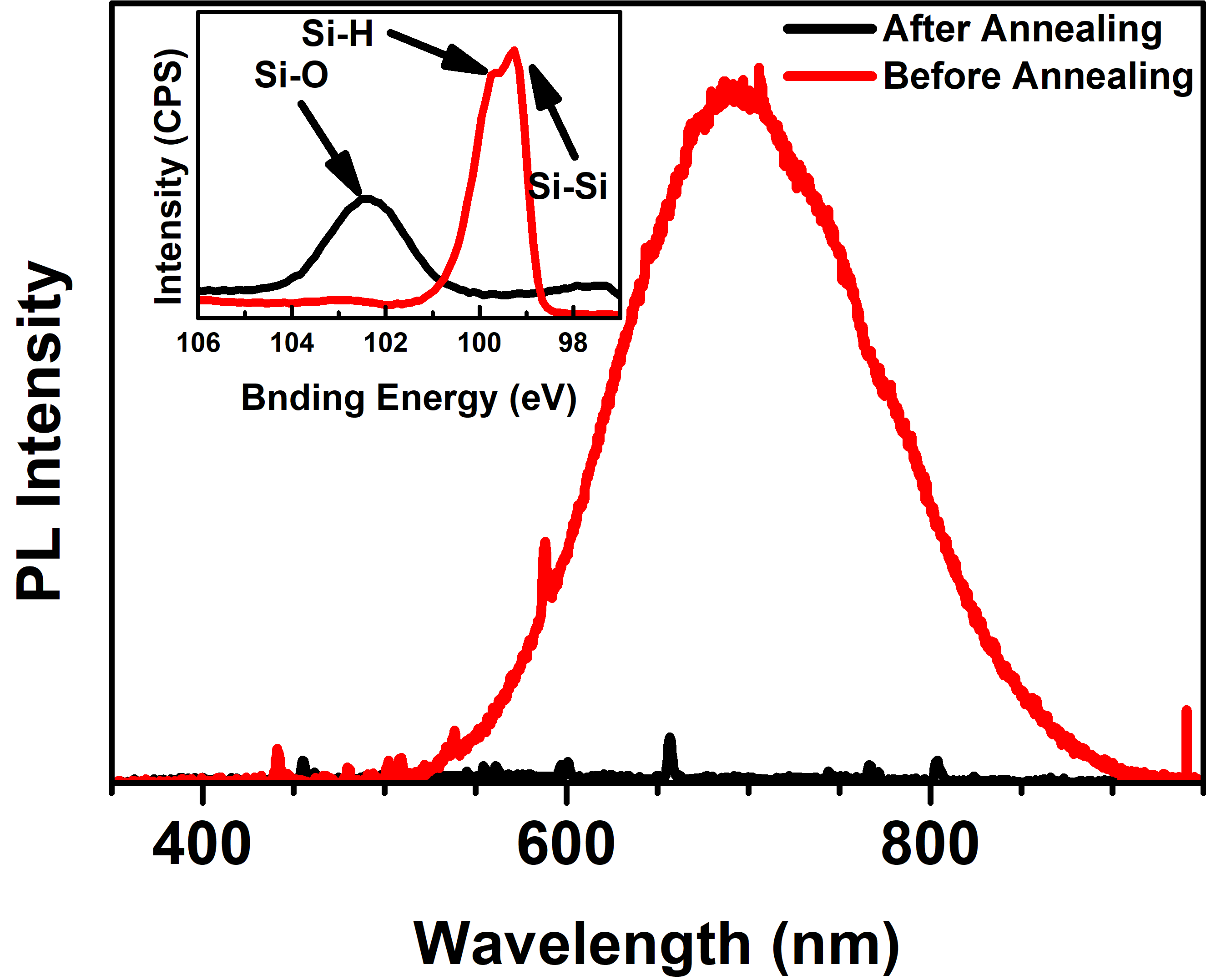


**Fig. S6:** PL emission spectra of pSi before and after thermal annealing at 500 ^o^C for 60 min. In the inset, the XPS spectra of *Si2p* of the samples are presented.

The as-prepared pSi before annealing exhibits orange/red emission (excitation with a 325 nm laser beam) centered at around 690 nm, which is, however, quenched and eliminated after thermal annealing apart from some individual sharp lines (Fig. S6). This difference can be explained by the change of the chemical composition of the pSi, as revealed by the XPS *Si 2p* core level spectra before and after the thermal annealing (Fig. S6-inset). Before the thermal annealing, a twin peak is observed. This peak can be deconvoluted in two different contributions that correspond to Si-Si and Si-H bonds, respectively. After the thermal annealing of the pSi, the *Si 2p* is characteristic of Si-O bonds. Thus, total oxidation of pSi occurs during the thermal annealing step and consequently, the pSi emission vanishes.

**Fig. S7:** XRD patterns of electrodeposited Zn before the annealing to form ZnO on pSi with pores <50 nm (red line) and on polished Si (100) wafer (blue line); the open (100) and (101) planes dominate the Zn deposited on pSi, as depicted by the red sketches of Zn’s unit cell, while the close packing (002) planes dominate the Zn deposited on the polished Si, as depicted by the blue sketch of the Zn’s unit cell.
